# Expression of a Humanized Viral 2A-Mediated *lux* Operon Efficiently Generates Autonomous Bioluminescence in Human Cells

**DOI:** 10.1371/journal.pone.0096347

**Published:** 2014-05-02

**Authors:** Tingting Xu, Steven Ripp, Gary S. Sayler, Dan M. Close

**Affiliations:** 1 The Joint Institute for Biological Sciences, Oak Ridge National Laboratory, Oak Ridge, Tennessee, United States of America; 2 490 BioTech, Inc., Knoxville, Tennessee, United States of America; University of Texas Southwestern Medical Center, United States of America

## Abstract

**Background:**

Expression of autonomous bioluminescence from human cells was previously reported to be impossible, suggesting that all bioluminescent-based mammalian reporter systems must therefore require application of a potentially influential chemical substrate. While this was disproven when the bacterial luciferase (*lux*) cassette was demonstrated to function in a human cell, its expression required multiple genetic constructs, was functional in only a single cell type, and generated a significantly reduced signal compared to substrate-requiring systems. Here we investigate the use of a humanized, viral 2A-linked *lux* genetic architecture for the efficient introduction of an autobioluminescent phenotype across a variety of human cell lines.

**Methodology/Principal Findings:**

The *lux* cassette was codon optimized and assembled into a synthetic human expression operon using viral 2A elements as linker regions. Human kidney, breast cancer, and colorectal cancer cell lines were both transiently and stably transfected with the humanized operon and the resulting autobioluminescent phenotype was evaluated using common imaging instrumentation. Autobioluminescent cells were screened for cytotoxic effects resulting from *lux* expression and their utility as bioreporters was evaluated through the demonstration of repeated monitoring of single populations over a prolonged period using both a modified E-SCREEN assay for estrogen detection and a classical cytotoxic compound detection assay for the antibiotic Zeocin. Furthermore, the use of self-directed bioluminescent initiation in response to target detection was assessed to determine its amenability towards deployment as fully autonomous sensors. In all cases, bioluminescent measurements were supported with traditional genetic and transcriptomic evaluations.

**Conclusions/Significance:**

Our results demonstrate that the viral 2A-linked, humanized *lux* genetic architecture successfully produced autobioluminescent phenotypes in all cell lines tested without the induction of cytotoxicity. This autobioluminescent phenotype allowed for repeated interrogation of populations and self-directed control of bioluminescent activation with detection limits and EC_50_ values similar to traditional reporter systems, making the autobioluminescent cells amenable to automated monitoring and significantly reducing the time and cost required to perform bioluminescent workflows.

## Introduction

The use of high signal to noise bioluminescent sensor technology is quickly replacing traditional fluorescent sensor technologies for research and pre-clinical applications. This trend has been supported by a substantial increase in bioluminescent sensor related publications in the past two decades and by a doubling in funding submissions to the National Cancer Institute between 1999 and 2007 that requested optical imaging equipment over conventional MRI or PET medical imagers [1]. However, despite its widespread adoption, this technology has remained stagnant and forced the optical imaging community to rely almost exclusively on the bioluminescent firefly luciferase gene (*luc*), which was first cloned and used as a sensor target in 1986 [2]. Despite improvements over the last decade that have enhanced *luc* gene expression [3], or the introduction of similarly functioning Renilla (introduced in 1991) [4] and Gaussia (introduced in 2002) [5] luciferase sensor systems, these technologies remain limited due to their requisite administration of a light activating chemical substrate (luciferin) that must be repeatedly purchased, is sensitive to light, oxygen, high pH exposure, or repeated freeze/thaw cycles, and, when applied concurrent with cellular lysis as is common in most commercial luciferase assay kits, yields only single time point data.

For these reasons, we have focused on the development of the bacterial luciferase (*lux*) sensor system, as it is the only known bioluminescent system capable of autonomously producing both its luciferase and associated luciferin generating protein products without exogenous investigator interaction. This is possible because, unlike alternate bioluminescent reporter systems such as firefly, Renilla, or Gaussia luciferase, the *lux* system consists of a series of six genes (*luxCDABE* and *frp*) that synergistically combine to produce both a luciferase, as well as a secondary protein complex that generates and recycles its required luciferin substrates from components found endogenously in the host cell microenvironment [6] (Figure S1).

The luciferase component is a heterodimer supplied by the products of the *luxA* and *luxB* genes, while the *luxC*, *luxD*, and *luxE* genes are responsible for encoding a reductase, a synthase, and a transferase, respectively. These gene products form a tetrameric trimer that acts as a cohesive unit to convert and recycle the required aliphatic aldehyde substrate from intracellular components originally bound for membrane biogenesis [7]. The *frp* gene, which is not found in all species, encodes a flavin reductase that is used to shift the intracellular FMN:FMNH_2_ balance to a more reduced state in order to supply the remaining FMNH_2_ co-substrate [6], which has been suggested to act primarily in a structural role through its attachment in its anionic state (FMNH^•^) rather than as a reduction partner as it is traditionally employed [8], although the end result is similar with its eventual oxidation back to FMN. When coordinately expressed, this system allows cells expressing the *lux* genes to assume an autonomously bioluminescent phenotype at a peak wavelength of 490 nm under the reaction scheme:

FMNH_2_+RCHO+O_2_ → FMN+H_2_O+RCOOH+*hv*_490 nm_

The preferred substrate for this reaction is suggested to be myristyl aldehyde, which is generated from myristyl-ACP in the native bacterial system, but can also be sourced from myristyl-CoA, which may be more prevalent in eukaryotes [6]. In addition, alternative chain length fatty acids can be similarly utilized by the LuxCDE protein complex, and the substrate used does not appear to have any effect on the wavelength of light produced [9].

Despite previous reports stating that the *lux* system was not capable of functioning in eukaryotic organisms [10], we have previously demonstrated that this is not the case by generating a substrate independent bioluminescent phenotype in a human cell line using *lux* technology, but were only able to achieve expression in a single cell line and were severely limited in signal strength relative to alternative, substrate-requiring luciferase systems [11], [12]. Recognizing that these deficiencies were limiting towards the potential use and growth of the *lux* system as an alternative to currently available bioluminescent systems, we here demonstrate a complete re-engineering of the system to streamline its introduction into human cells, increase the efficiency of new cell line development, increase bioluminescent output signal, and provide an improved platform for the development of target-specific bioreporters that can modulate light output in response to chemical detection.

## Results

### Development of a Humanized *lux* Operon

**Figure 1:**
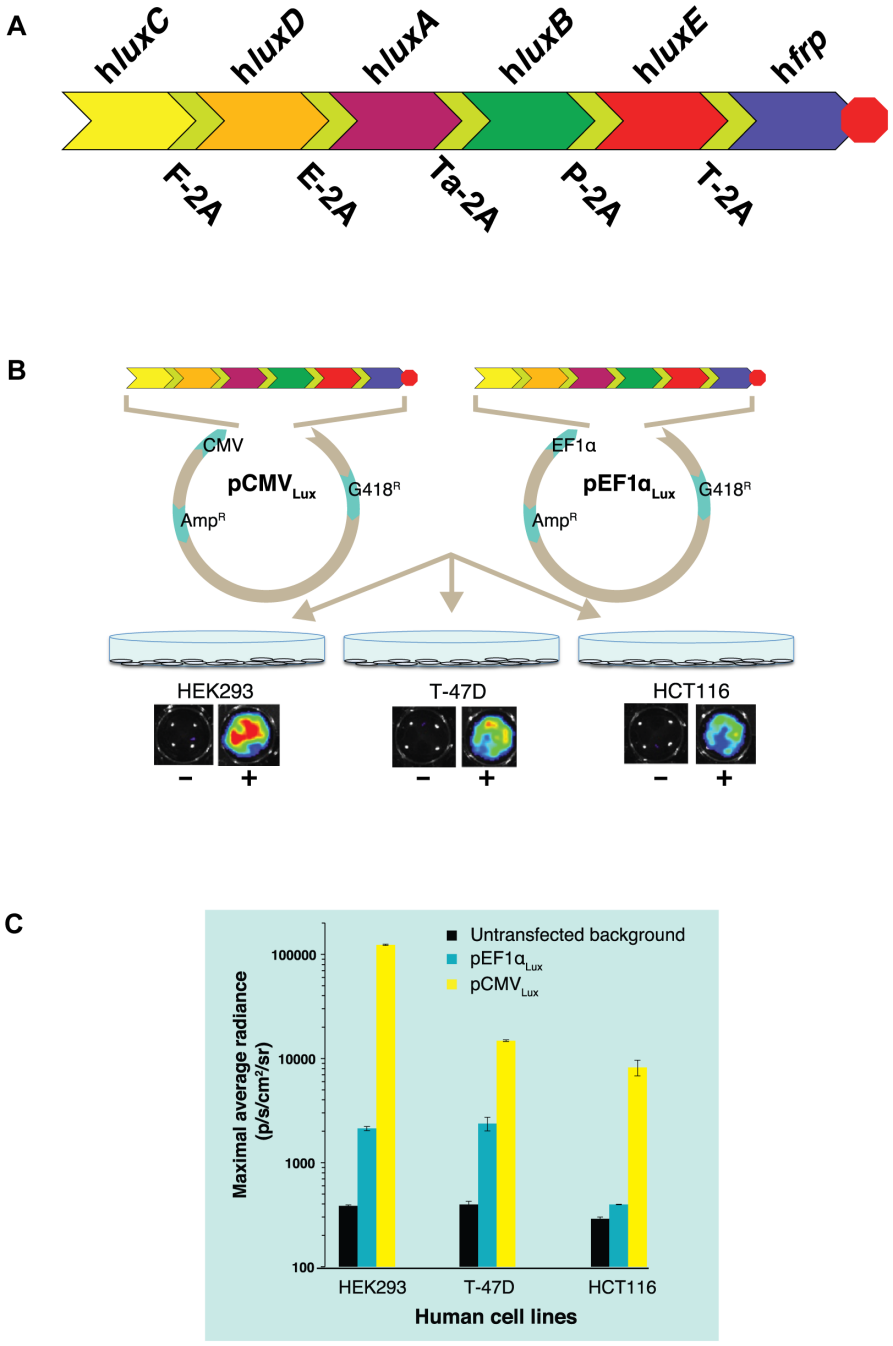
Schematic illustrating the construction and evaluation of autobioluminescent human cell lines. (A) The *luxCDABE* genes of *Photorhabdus luminescens* and the *frp* oxidoreductase gene of *Vibrio campbellii* were codon optimized for expression in human cells and linked using unique viral 2A elements. Intervening stop codons were removed, leaving only the final stop codon (red octagon) at the 3′ end of the *frp* gene to create a single, continuous ORF. (B) The human optimized *lux* operon was then cloned under the control of either a CMV or EF1α promoter and transiently transfected into HEK293 (kidney), T-47D (breast cancer), and HCT116 (colorectal cancer) human cell lines, resulting in an autobioluminescent phenotype for each cell type. (C) Expression of the 2A-linked *lux* cassette under control of the CMV promoter produced significantly (*p*≤0.05) higher maximal autobioluminescent output levels in each of the cell lines relative to EF1α-based expression, making it an improved choice for the development of constitutively autobioluminescent cell lines. The CMV-based cassette was then stably transfected into the same parent cell lines and the clonal lines with the highest levels of autobioluminescent output were selected for further analysis.

Stable transfection of HEK293 cells using the previously described [12] two vector *lux* expression system resulted in inefficient generation of autobioluminescent phenotypes, with a successful transfection efficiency of <1%. To improve the efficiency of the transfection procedure, the humanized *lux* cassette was redesigned to reduce its overall size and place all of the required genes on a single plasmid vector. This was accomplished by replacing the previously utilized internal ribosomal entry site (IRES) linker regions with viral 2A linker regions containing genetically unique upstream glycine/serine flexible linkers (Table S1), as this strategy has been demonstrated to increase autocleavage efficiency during sequence translation [13]. The intervening stop codons from each of the *lux* genes were then removed to generate a single, continuous open reading frame to allow for coordinated expression of the full *lux* cassette from a single promoter (Figure 1A). This strategy reduced the overall size of the required construct from 7.3 kb to 6.7 kb, eliminated the incorporation of large (>500 bp) repeated sequences, and permitted selection to occur using a single antibiotic selection marker.

This newly designed *lux* expression architecture was placed under the control of a cytomegalovirus (CMV) promoter to create the construct pCMV_Lux_ and mimic the regulatory control of the previous two vector system. When transfected into the same parent HEK293 cell line, the transfection efficiency of the pCMV_Lux_ vector significantly increased (*p*≤0.05) to ∼50% and, similarly, autobioluminescent output increased from an average of 3.51×10^4^ p/s/well to 3.89 ×10^6^ p/s/well for the same number of transfected cells.

### Transient Transfection of Human Cell Lines

With evidence suggesting the new human optimized *lux* operon genetic architecture could efficiently generate autobioluminescent phenotypes in the HEK293 cell line, a new vector was then developed that placed the humanized *lux* cassette under the control of an endogenous human elongation factor 1α (EF1α) promoter (Figure 1B), as this promoter has been suggested to provide increased gene expression relative to CMV in HEK293 cells [14]. This new construct was named pEF1α_Lux_. Both the pCMV_Lux_ and pEF1α_Lux_ vectors were then transiently transfected into a variety of human cell types including kidney (HEK293), breast cancer (T47D) and colorectal cancer (HCT116) cells as described in material and methods.

Both constructs (pCMV_Lux_ and pEF1α_Lux_) were capable of successfully producing a transient autobioluminescent phenotype across all lines tested. Surprisingly, in all cases CMV-driven expression resulted in superior bioluminescent output compared to EF1α-driven expression, despite that no significant difference in transfection efficiency was found using a co-transfected firefly luciferase reporter as an internal control (*p* = 0.368) (Figure 1C). This was in contradiction to previous reports that the EF1α promoter is capable of driving increased expression in HEK293 cells relative to CMV [14].

### Transcript Analysis of Viral 2A-Linked *lux* Gene Expression

To verify that the new single promoter/viral 2A expression strategy was sufficient for coordinated expression of all six required *lux* genes, and to determine if increased *lux* gene transcript levels due to CMV-driven expression was responsible for the increased autobioluminescent phenotype relative to EF1α-driven expression, an analysis of *lux* gene mRNA transcript levels was performed using qRT-PCR. This analysis revealed that all six *lux* cassette genes were being expressed from both the EF1α and CMV promoters, but that the expression level of each gene was greater under the control of the CMV promoter (Figure S2 and Table S2). On average, expression of each of the *lux* genes was 7.62 (±0.69) –fold greater when placed under the control of the CMV promoter. Notably, use of the CMV promoter lead to a prevalent increase in expression of the genes most distal to the promoter, with the *luxE* gene (∼4.8 kb distal to CMV) increasing 8.09-fold, and the *frp* gene (∼6.0 kb distal to CMV) increasing 9.72-fold.

### Development of Constitutively Autobioluminescent Human Cell Lines

Since both the CCD camera-based autobioluminescent readings and the transcript level analysis suggested the CMV-mediated pCMV_Lux_ vector was capable of producing a superior autobioluminescent phenotype, this vector was selected for the generation of stably transfected cell lines. Just as with the transient transfection, the single vector-mediated *lux* expression system was capable of efficiently producing an autobioluminescent phenotype in each of the cell lines tested, and allowed for improved selection efficiency due to the need for only a single selective marker. Each of the stably transfected autobioluminescent cell lines maintained its phenotype across all generations assayed and could be assayed repeatedly for bioluminescent production at any chosen interval without any external stimulation required to elicit signal generation. The HEK293 cell line, which produced the highest level of autobioluminescent output under transient expression conditions (Figure 1C), could be detected using either CCD camera-based or PMT-based plate readers with a 1 second exposure (Figure S3). Detection was possible in multiple plate formats down to 1536 well and correlated strongly (R^2^≥0.982) with total cell number across all cell types (Figure S4), although detection was only possible down to the level of approximately 1×10^3^ cells (Figure S3) when grown in the maximum tested surface area of a 24 well plate (1.9 cm^2^).

### Evaluation of Metabolic Activity Level Dynamics Resulting From Autonomous Bioluminescent Expression

Similar to how the better characterized *luc* luciferase system leverages endogenous cellular O_2_ and ATP for the generation of its bioluminescent signal, the *lux* system is dependent on its utilization of cellular O_2_ and FMN/FMNH_2_, and additionally produces an aldehyde substrate that is potentially cytotoxic. To determine the extent of any cytotoxic effects induced though expression of the autobioluminescent phenotype, metabolic activity levels were evaluated using the commercially available CellTiter-Glo assay to compare the ATP levels between wild type and autobioluminescent cell lines as an indicator of metabolic activity. Both wild type and autobioluminescent HEK293 and HCT116 cells displayed strong correlations between cell number and metabolic activity both immediately after plating and following 24 h of growth (R^2^≥0.979). Similarly, the correlations between metabolic activity levels of both wild type and autobioluminescent HEK293 (Figure S5A) and wild type and autobioluminescent HCT116 (Figure S5B) were strong (R^2^≥0.997), with only the 24 h post-plating measurement of 1000 HEK293 cells and the 0 h post plating of 1000 HCT116 cells displaying low, but significant differences (*p*≤0.05) in ATP levels, although neither remained significant at a *p* = 0.01 level (Table S3).

### Evaluation of Oxidative Stress Resulting From Autonomous Bioluminescent Expression

To further evaluate potential cytotoxic effects stemming from the presence of reactive oxygen species generated during the autobioluminescent process, wild type and autobioluminescent cells were screened for the reactive oxygen marker H_2_O_2_ using the commercially available ROS-Glo assay. Total reactive oxygen species detection levels correlated strongly between the wild type and autobioluminescent cells for both HEK293 (R^2^ = 0.951) and HCT116 (R^2^ = 0.970) for all cell population sizes assayed (Figure S6). For both the wild type and autobioluminescent cells, higher reactive oxygen species levels were detected in the HCT116 samples than in the HEK293 samples. These results indicate that the activation of an autobioluminescent phenotype does not significantly increase the presence of reactive oxygen species in either of the cell lines tested.

### Comparison of Treatment-Induced Cytotoxic Effects Between Wild Type and Autobioluminescent Cell Lines

Although the previous results suggested that autobioluminescent cells do not display enhanced autocytotoxic effects under routine growth conditions, the possibility remained that these increased cytotoxic effects could manifest under the adverse conditions generated during toxicological screening. To investigate this possibility, both wild type and autobioluminescent cells were treated with increasing doses of Zeocin, a cytotoxic compound commonly employed to kill these cell types upon treatment. Both HEK293 (Figures S7A and S7B) and HCT116 (Figures S7C and S7D) cells responded similarly to increasing Zeocin treatment levels, with cell death increasing in response to increased Zeocin concentrations. Reductions in cellular viability as measured using a standard MTT assay were within error (*p*>0.05) for all Zeocin concentrations tested at both 24 and 48 h post treatment, with the exception of the 200 µg/ml treatment of HEK293 cells as measured 48 h after Zeocin application (p = 0.01). In this case, a significantly higher number of viable wild type cells were observed compared to similarly treated autobioluminescent cells, although it is not known what factors lead to this observation only under these specific conditions.

### Comparison of Treatment-Induced Cytotoxic Effects Between *luc*-Expressing and *lux*-Expressing Cell Lines

Comparisons were performed to determine if the cytotoxic effects resulting from Zeocin treatment manifest similarly between traditional *luc*-expressing cell lines and autobioluminescent *lux*-expressing cell lines. MTT assays comparing cell viability following 24 h Zeocin treatment indicated that cytotoxic effects were similar between cells expressing the two different bioluminescent systems, with *luc* and *lux*-expressing HEK293 cell viability measurements correlating at an R^2^ value of 0.963 and HCT116 cell viability measurements correlating with an R^2^ value of 0.991. Correlation between bioluminescent output and viability was also strong for the HCT116 cells, with autobioluminescent output correlating with MTT viability measurements at an R^2^ value of 0.926 (compared to an R^2^ value of 0.997 for *luc*-expressing cells) (Figure S8A). The correlation between autobioluminescent output and MTT viability measurements for autobioluminescent HEK293 cells (Figure S8B) were lower (R^2^ = 0.687), despite a similar correlation being maintained by the *luc*-expressing cells (R^2^ = 0.978).

### Detection of 17β-Estradiol Using Continuously Autobioluminescent T47D Cells

**Figure 2:**
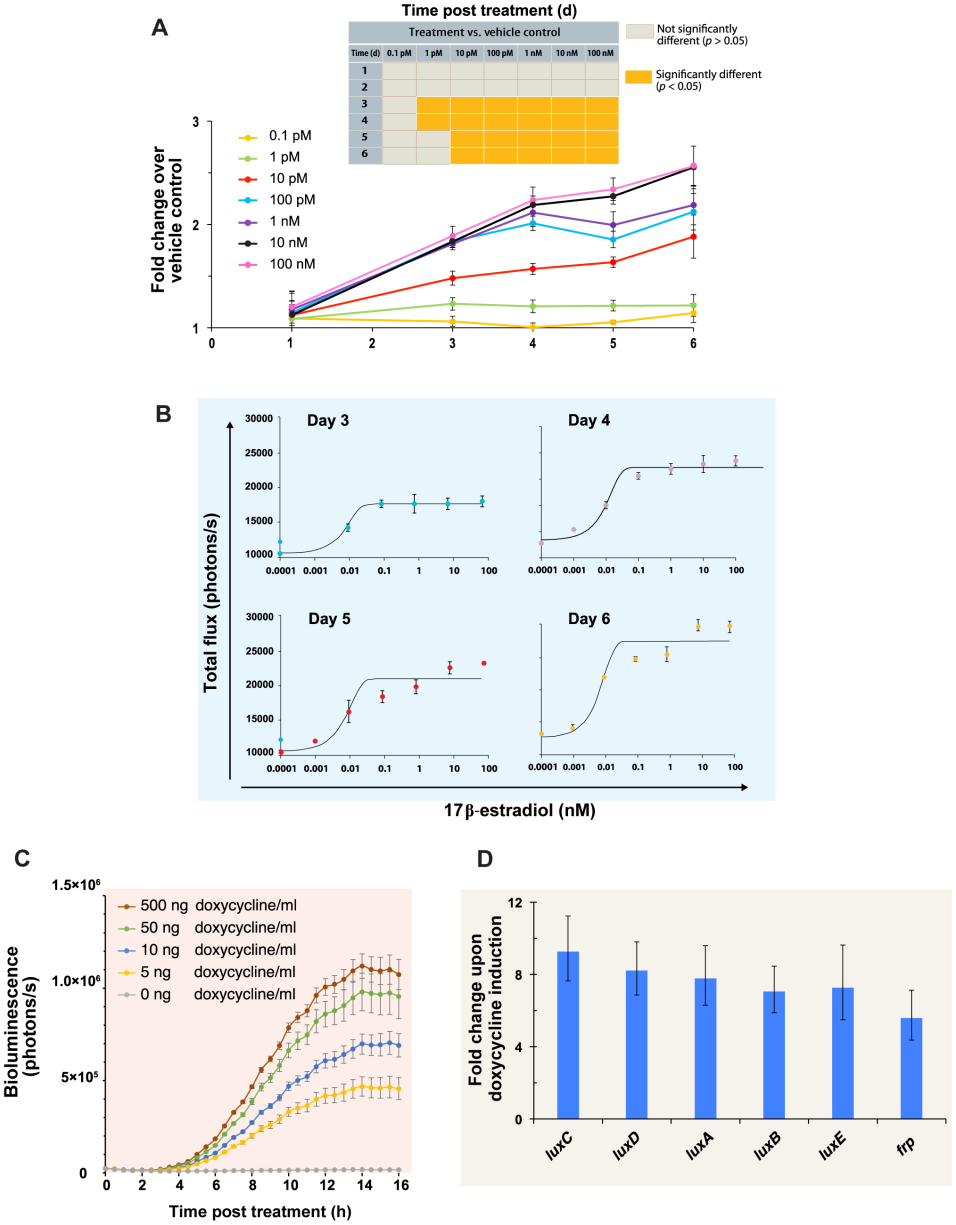
Application of autobioluminescent human cells as target-responsive bioreporters. (A) Human T-47D breast cancer cells naturally express estrogen receptors and proliferate in response to increased exposure to estrogenic compounds. When treated with 17β-estradiol, T-47D cells expressing the pCMV_Lux_ plasmid displayed increased growth rates compared to untreated control cells by day 3 for all treatments ≥1 pM, although this trend was only maintained throughout the full 6 day assay period at treatment levels ≥10 pM (inset). (B) A dose-response relationship was determined between 17β-estradiol and autobioluminescence, with an EC_50_ value (10 pM) similar to that of traditional proliferation assays and alternative bioluminescent reporter systems. (C) When the human optimized *lux* operon was placed under the control of a doxycycline responsive Tet-On promoter, HEK293 cells could autoreport detected concentrations ≥5 ng/ml. (D) An up-regulation of *lux* gene mRNA transcript levels was observed upon doxycycline treatment, indicating that the increase in autobioluminescent production was due to activation of the Tet-On promoter and not the result of non-specific treatment effects.

To determine if the correlation between autobioluminescence and cell number, combined with the stable, autonomous nature of *lux*-based bioluminescence, could be leveraged to automate routine bioluminescent workflows, an estrogen detecting E-SCREEN [15] assay was prepared by treating stably transfected, autonomously bioluminescent T-47D breast cancer cells with increasing levels of 17β-estradiol. Over the course of six days, the same cells were repeatedly screened for autobioluminescent output without the need for substrate application. Changes in population size due to increased growth rate were detected by day three for all treatment concentrations ≥1 pM (Figure 2A). Dose response kinetics were observed on each day that demonstrated a significant change in growth rate and an EC_50_ value of 10 pM was calculated, which is consistent with results obtained using a traditional proliferation assay [16] or alternative luciferase reporter-based assays (6–12 pM) [17], [18] (Figure 2B). Under this assay format it is possible to automate routine monitoring of cellular population dynamics, reducing the amount of hands on time and external intervention required by negating the need for repeated applications of a chemical substrate.

### Comparison of Cytotoxic Compound Detection Using Autobioluminescent and Substrate-Induced Bioluminescent Cell Lines

To determine if there were differences in the ability of *lux*-based autobioluminescent and *luc*-based substrate-dependent-bioluminescent cell lines for the detection of cytotoxic compounds, identical numbers of *lux* and *luc* transfected HEK293 and HCT116 cells were plated and similarly exposed to increasing concentrations of Zeocin. Cells were then repeatedly assayed for bioluminescent output every 6 h for a 24 h period. Under these detection conditions, the *luc* cell-based reporters performed identically regardless of the cell type employed, with both HEK293 and HCT116 reporter cells demonstrating consistent significantly (*p*≤0.05) decreased bioluminescent signals compared to untreated control cells at 18 h post treatment (Table S4). Autobioluminescent HEK293 cells were capable of consistently identifying cytotoxic effects at the 200 and 800 µg/ml treatment levels beginning at 12 h post treatment, with detection of all treatment levels beginning at 18 h post treatment. Autobioluminescent HCT116 cells, however, did not display positive detections for any of the treatment levels throughout the 24 h duration of the assay (Table S4).

### Autonomous Detection of Doxycycline Using A Self-Regulated HEK293 Bioreporter Cell Line

Fortuitously, with the full 2A-linked *lux* operon now under the control of a single promoter, we determined that it should be conversely possible to coordinately regulate expression of the genes simultaneously and allow for the development of fully autonomous human cell bioreporters that instead initiate bioluminescent production in response to target detection. To investigate if this approach was feasible, the CMV promoter of pCMV_Lux_ was replaced with a tetracycline responsive TET-On promoter and cells were exposed to doxycycline to generate an autobioluminescent detection reporter signal. Significant (*p*≤0.05) increases in autobioluminescence were detected by 2.5 h following doxycycline treatment for all treatment levels, and output levels continued to increase with increasing exposure time (Figure 2C). Analysis of mRNA transcript levels indicated that this increase was related to an increase in *lux* gene mRNA copy number (Figure 2D), with significant (*p*≤0.05) increases detected in all the *lux* genes (Table S5). The ability of this expression strategy to produce detectable autobioluminescent signals under the control of three different promoters (EF1α, CMV, and Tet-On) suggests that it can be adapted to function as a fully autonomous detection system for a wide variety of compounds or cellular events with known regulatable promoter systems, however, further work is required to determine if additional promoters are capable of driving expression across the full expanse of the 2A-linked operon.

## Discussion

The *lux* operon provides a unique means for the generation of bioluminescence without requisite substrate administration because it is comprised of six genes (*luxCDABEfrp*) that, when expressed concurrently, produce a 490 nm bioluminescent signal [6] (Figure S1). Previous attempts have been made to express the full *lux* operon within a eukaryotic cellular host, although these have been met with limited success and, in general, only individual genes have been recognized and transcribed by these cells [19]–[21]. The major challenges preventing efficient expression of the full operon have hinged on its large size, multi-gene organization, and the potential for cytotoxicity stemming from either the redirection of metabolic flux into production of the self-generated aldehyde substrate, or the interaction of that substrate with endogenous cellular components.

Potentially cytotoxic effects resulting from autobioluminescent expression were evaluated by monitoring metabolic activity levels, detecting reactive oxygen species generation, and through general cell viability testing. The viability of *lux*-expressing autobioluminescent cells was similar to that of traditional *luc*-expressing cells under both routine growth conditions and during cytotoxic compound exposure, which was expected given that the basal metabolic activity of the cells was observed to remain relatively unchanged compared to their wild type parental cell lines (Figure S5 and Table S3). Interestingly, cells expressing the *lux* system displayed a decrease in measureable reactive oxygen species compared to their wild type counterparts (Figure S6), suggesting that the presence of the *lux* expression system may be capable of reducing oxidative stress through its routine function.

In contrast, we observed that the more pressing hurdles preventing expression of the *lux* system have been its large size and multi-gene organization, the combination of which required co-expression of multiple plasmids and the use of IRES elements to link together individual genes for pseudo-polycistronic expression. The use of these IRES elements was found to be particularly limiting since their mechanism of action relies on the formation of secondary structure in the mRNA transcript to recruit additional ribosomes that initiate cap-independent translation of the downstream gene [22]. Under previously successful expression conditions, the resulting decreased transcription levels of the downstream gene prohibited the expression of more than two genes from a single promoter [12], requiring three total promoters be used to express the full six gene cassette, and introducing a need for both the incorporation of repetitive DNA sequences that were prone to recombination and the division of the cassette across multiple vectors that needed to be cotransfected and coordinately expressed in order to generate an autobioluminescent phenotype.

Viral 2A elements, in contrast to IRES elements, perform the same ultimate function of allowing multiple proteins to be translated from a single transcript, but use a mechanism of action that is more amenable to humanized polycistronic expression. This is possible because the steric hindrance imparted on the exit tunnel of the ribosome by the newly synthesized 2A element peptide sequence causes a “skipping” of the last peptide bond at the C terminus of the 2A sequence. Despite this missing bond, the ribosome is able to continue translation, creating a second, independent protein product. These sequences, because they do not rely on secondary structure formation, are therefore much shorter than the IRES elements sequences, are not as prone to reducing the efficiency of the transcription and translation processes [23], [24], and have previously been demonstrated as an efficient means for the expression of multiple protein products in eukaryotic systems [25]–[28].

The result of this size reduction and increased transcriptional efficiency has therefore allowed the full *lux* cassette to be placed under the control of a single promoter and expressed off of one vector. Forgoing the need to cotransfect and coselect for multiple constructs while screening for clones that randomly integrated each of the separate *lux* gene sections into genomic locations capable of coordinating their expression in ratios sufficient to generate a usable autobioluminescent signal has significantly increased the efficiency of new cell line development and lead to an increase in autobioluminescent output levels. The new system is now much more similar to that of traditional *luc* reporters [29], which can be easily expressed as a single gene.

However, one significant difference remaining between the *lux* and *luc* systems is that of their resultant output wavelengths (490 nm and 562 nm, respectively). While the blue-shifted emission wavelength of the *lux* system is not ideal for expression in tissue, it has been demonstrated that the resultant autobioluminescent signal can still be efficiently detected using common bioluminescent imaging equipment (Figure S3), and that the autonomous nature of *lux*-based bioluminescence provides an increased degree of utility compared to substrate-requiring luciferases by allowing for continuous or automated analysis of the same samples repeatedly without re-administration of a luciferin substrate. This has been demonstrated through the development of a substrate-free E-SCREEN assay using an autobioluminescent T47D human breast cancer cell line. Because the E-SCREEN assay tracks the proliferation of estrogen sensitive cells, and because autobioluminescent production is tightly correlated with population size in this cell line (R^2^ = 0.999) (Figure S4), populations of these autobioluminescent cells can be continuously or repeatedly monitored to track their growth in response to estrogenic compound treatment. With no investigator intervention or cellular destruction required to interrogate the resultant autobioluminescent signal, this process is therefore highly amenable to automation and can reduce both the cost and hands on time required [30].

This advantage was similarly leveraged throughout the course of our cytotoxic treatment assays, where forgoing the need to administer a luciferin treatment prior to imaging resulted in shorter assay preparation times and higher amenability to automation by eliminating the need for investigator interaction prior to each of the assayed time points. However, the trade off for this increased ease of use was manifest as a decrease in the bioluminescent output of the *lux*-based cells relative to the *luc*-based cells. While this decrease in signal to noise ratio did not adversely alter cytotoxic effect detection in the HEK293 cell line (Table S4), the relatively decreased output of the autobioluminescent HCT116 cell line was not capable of generating a baseline bioluminescent signal strong enough to permit differentiation of the decreased cytotoxic detection signals at *p* values less than 0.05. However, based on the similar performance of the *lux* and *luc*-based HEK293 cell lines and the lack of autocytotoxic effects observed in these HCT116 cells (Figures S5B, S6B, and S8A), it is believed that further development of this line to increase autobioluminescent output could overcome this deficiency.

Another advantage of this redesigned *lux* operon is that the use of a single promoter construct for coordinate regulation of the full cassette increases its utility as a fully autonomous human cell bioreporter. Previous reports based on mathematical modeling have suggested that regulation of only a subset of the *lux* genes would be required for reporter development [31], however, this approach would require the utilization of multiple promoter sequences in a manner similar to the multiple vector approach and could therefore affect both the efficiency of new cell line development and autobioluminescent output levels. In order to avoid these potential complications, a doxycycline-specific bioreporter was instead developed by replacing the constitutive CMV promoter with a Tet-On regulatory region to place control of the full operon under a single switch.

Using this approach, it was possible to coordinately regulate expression of the full *lux* cassette simultaneously, and achieve significant increases in autobioluminescent production within 2.5 h following doxycycline application (Figure 2C). The Tet-On regulatory system was capable of significantly increasing transcription of each of the *lux* genes by an average of 7.52 (±0.50)-fold, although expression levels decreased for each gene as they became increasingly distal to the promoter (Table S5). The Tet-On system was chosen because it represents a common method for evaluating the effectiveness of a reporter system as it has previously been shown to be effective for expression of a wide array of reporter genes across multiple mammalian cell lines [32], allowing for the facile comparison of reporter function with previously published models. This comparison revealed that detection limits were similar to previously published results, but that the minimum time until detection was longer [33], [34], possibly due to the need for expression of multiple genetic elements, their translation and formation into multimeric constructs, and subsequent interaction with endogenous cellular processes to generate the required luciferin components and co-substrates.

Despite this delay in detection time, the fully autonomous nature of detection and subsequent signal generation retains the potential for increased utility relative to luciferin-requiring or fluorescent bioreporters in that these autobioluminescent reporters can be monitored continuously in an automated fashion to determine the precise onset of signal generation. Furthermore, unlike luciferin-requiring reporter systems, these autobioluminescent bioreporters can be continuously interrogated without investigator interaction to initiate signal generation, allowing for the assessment of both signal duration and intensity dynamics from a single sample and, unlike fluorescent systems, they are not subject to photobleaching or tissue autofluorescence resulting from exposure to UV light. The fact that the impetus for signal generation is self generated also removes concerns arising from UV-mediated cellular damage or potential influential effects of chemical luciferin treatment, however, there still exists a concern that the internally generated aldehyde substrate could become cytotoxic if accumulated to high levels [35]. We, however, did not observe any significant cytotoxicity that could be attributed to aldehyde production or retention using either the single vector or two vector *lux* expression system [12]. This observation, combined with the observed similar metabolic activity levels, a reduction in reactive oxygen species availability, and the similar viability dynamics in response to toxicant exposure of the autobioluminescent cell lines relative to their wild type and *luc*-expressing counterparts, suggests that these concerns may be unfounded. However, it is worth noting that the decreased reactive oxygen species level in *lux*-expressing cells may indicate interference with normal reactive oxygen species-mediated processes such as intracellular signaling, presenting a potential disadvantage of the *lux* reporter system.

While this demonstration of how the full *lux* operon can be adapted for efficient expression in human cells can provide a springboard for its further use as an autonomous, real-time reporter system, it is especially relevant that its distinct wavelength output and autonomous production potential make it well suited for use alongside existing fluorescent and bioluminescent proteins. By employing the humanized *lux* system in tandem with existing reporter systems it may be possible to obtain more data than was previously possible from dual luciferase systems, and to do so using existing protocols and equipment. Similarly, just as currently available fluorescent and bioluminescent reporter systems have enjoyed modifications to expand their utility and enhance their functionality, the novel nature of the humanized *lux* system suggests that it too may be subjected to these evolutions as its use becomes more widespread, making it a more attractive tool for use as an optical imaging target.

## Materials and Methods

### Bacterial Strain maintenance and growth

*Escherichia coli* cells were routinely grown in Luria Bertani (LB) broth with continuous shaking (200 rpm) at 37°C. When required, kanamycin or ampicillin was used at final concentrations of 40 and 100 µg/ml, respectfully, for selection of plasmid containing cells.

### pEF1α_Lux_ expression plasmid construction

The wild type sequences for the *luxCDABE* genes from *Photorhabdus luminescens* (accession number M90093.1) and the *frp* gene from *Vibrio campbellii* (accession number U08996.1) were downloaded from NCBI. Sequences were assembled *in silico* with glycine/serine flexible linkers and intervening viral 2A elements as described in Figure 1A and Table S1. Stop codons were removed from all genes with the exception of *frp* to create a single open reading frame of 6,705 bp, which was then codon optimized for human expression and synthetically assembled by GeneArt, Inc. The humanized *lux* operon was then cloned into the multiple cloning site (MCS) of the pEF-myc-nuc vector (Life Technologies) using the 5′ Nco I and 3′ Xba I restriction sites. A promoter-less plasmid, pLux, was also constructed by replacing the EF1α promoter-containing Acc65 I/Nco I fragment from the pEF1α_Lux_ plasmid with a MCS DNA sequence and a CMV intermediate early intron sequence to facilitate downstream cloning applications.

### pCMV_Lux_ expression plasmid construction

The CMV promoter from the pIRES-bi plasmid (Clontech) was PCR amplified with added 5′ Mlu I and 3′ Sal I sites. The resulting 781 bp fragment was gel purified (Qiagen), digested with Mlu I and Sal I, and cloned into compatible restriction sites of the promoter-less pLux plasmid. The resulting construct (pCMV_Lux_, Figure 1B) was verified by sequencing on an ABI 3100 genetic analyzer (Applied Biosystems).

### pTETOnLux reporter plasmid construction

The pTRE-Tight-bi plasmid (Clontech) was digested with Acc65 I and Xba I. The 463 bp fragment representing the Tet-On regulatory region and CMV_mini_ promoter was gel purified (Qiagen) and cloned into compatible restriction sites of the promoter-less pLux plasmid. The resulting construct (pTetOn_Lux_) was verified by sequencing on an ABI 3100 genetic analyzer (Applied Biosystems).

### Cell culture

All cells were grown at 37°C, 5% CO_2_. HEK293 (ATCC # CRL-1573) cells were grown in DMEM media supplemented with 10% FBS, 0.01 mM non-essential amino acids (Life Technologies), 1X antibiotic-antimycotic (Life Technologies), and 1 mM sodium pyruvate (Life Technologies). T-47D (ATCC # HTB-133) cells were grown in RPMI-1640 (Hyclone) supplemented with 10% FBS, 0.01 mM non-essential amino acids, 1X antibiotic-antimycotic, and 1 mM sodium pyruvate. HCT116 (ATCC # CCL-247) cells were grown in McCoy's 5A media supplemented with 10% FBS, 0.01 mM non-essential amino acids, 1X antibiotic-antimycotic, and 1 mM sodium pyruvate. For all cell lines, the appropriate medium was refreshed every 3^rd^ day as required, and cells were passaged 1∶10 upon reaching 80% confluence.

### Transfection

For transient transfection, cells were co-transfected with the indicated *lux* cassette vector and a pGL4.5 vector (Promega) containing the *luc2* gene using Lipofectamine 2000 (Invitrogen). Autobioluminescence readings were taken 24 h post transfection and immediately followed with luciferin application and *luc2*-based bioluminescent readings for assessing transfection efficiency. For development of stable cell lines, transfections were performed as previously described, with the following modifications. Initial transfection was performed using a vector containing only the *luxAB* genes to develop genetically integrated targets for homologous recombination. Cells stably expressing the *luxAB* genes were subjected to hygromycin selection and *luxAB* expression was confirmed as previously described [19]. The clonal line with the highest level of *luxAB* expression was then re-transfected with a vector containing the full human optimized *lux* operon and subjected to G418 selection. Successfully established clonal lines were identified via bioluminescent imaging and the clonal line with the highest autobioluminescent output normalized to cell population size was selected for further analysis.

### Bioluminescent imaging

Cells were harvested and counted using a Scepter 2.0 handheld automated cell counter (Millipore). Equal numbers of cells were plated into triplicate wells of various opaque multiwell plates and transferred immediately to either a CCD-based IVIS Lumina imaging system (PerkinElmer) or a PMT-based Synergy 2 plate reader (BioTek). Autobioluminescent readings were then repeatedly taken at regular intervals. For multi-day imaging procedures, cells were maintained at 37°C, 5% CO_2_ between readings.

### mRNA transcript analysis

The transcript production levels of the *lux* genes were analyzed by qRT-PCR. Total RNA was extracted using an RNeasy Mini Kit (Qiagen) and treated with DNase I (Zymo Research). qRT-PCR reactions were performed using Brilliant II SYBR GREEN One-Step qRT-PCR Master Mix (Agilent) in 25 µl volumes. For each reaction, a total of 12.5 ng of RNA template was used for reverse transcription. All results were standardized to *β*-actin transcript levels. Relative gene expression in a given sample, or compared between two samples, was analyzed using the _Δ_C_q_ or _ΔΔ_C_q_ methods [36], [37], respectively.

### E-SCREEN assay

Approximately 1×10^4^ autobioluminescent T-47D cells were seeded into each well of a clear-bottom, tissue culture treated, black 24-well plate (Labnet International) in 1 ml growth medium and incubated under standard growth conditions for 24 h. After this time, the medium was removed, cells were washed once with 1 ml of PBS, and 1 ml of assay medium consisting of RPMI-1640 (Hyclone) supplemented with 10% charcoal/dextran-treated FBS, 0.01 mM non-essential amino acids, 1X antibiotic-antimycotic, and 0.01 mM sodium pyruvate. 17β-estradiol dissolved in HPLC grade ethanol was then supplemented to final concentrations of 0 pM (control), 0.1 pM, 1 pM, 10 pM, 100 pM, 1 nM, 10 nM, or 100 nM in triplicate wells. Solvent concentration remained constant at 0.1% (v/v) across all wells. Bioluminescence measurements were obtained using an IVIS Lumina imaging system with a 10 min integration time every 24 h for 6 days and cells were incubated at 37°C in a 5% CO_2_ environment between measurements.

### Doxycycline detection assay

HEK293 Tet-On 3G cells (Clontech) expressing the pTETOn_Lux_ plasmid were washed once with PBS, harvested, and counted using a Scepter 2.0 handheld automated cell counter (Millipore). Approximately 1×10^6^ cells/well were plated in 1 ml medium volumes in a black 24-well plate. Doxycycline was added at final concentrations of 0 (control), 5, 10, 50 or 500 ng/ml in triplicate wells. Bioluminescence measurements were obtained immediately after doxycycline induction using an IVIS Lumina imaging system with a 10 min integration time every 30 min for 16 h.

### Metabolic activity level determination assays

Autobioluminescent HEK293 or HCT116 cells stably transfected with pCMV_Lux_ vectors were seeded in triplicate into two 96 well plates at plating volumes of 0, 100, 250, 500, 1000, 2500, 5000, and 10000 cells/well in 100 µl volumes of medium. Identical numbers of untransfected wild type cells were similarly plated to act as positive controls. One plate each of the autobioluminescent pCMV_Lux_-expressing cells and wild type positive control cells was immediately subjected to ATP level measurement using the CellTiter-Glo assay (Promega) according to the manufacturer's instructions. Bioluminescent readings were obtained using a SynergyII plate reader (BioTek) with a 1 sec integration time and reported as relative light units. The second set of plates was incubated at 37°C and 5% CO_2_ for 24 h before processing with the CellTiter-Glo assay under identical conditions. This process was repeated three times to obtain three biological replicates, each consisting of three technical replicates. Relative light units from each plating density were averaged and the average values for each of the triplicate plates were used to determine standard errors of the mean for each assay.

### Reactive oxygen species detection assays

Autobioluminescent HCT116 cells stably transfected with the pCMV_Lux_ vector were seeded in triplicate into 96 well plates at plating volumes of 0, 100, 250, 500, 750, 1000, 2500, and 5000 cells/well in 80 µl volumes of medium. Identical numbers of untransfected wild type cells were similarly plated to act as positive controls. Due to the low observed values generated by wild type HEK293 cells, only platings of 0, 500, 1000, 5000, and 1000 wild type and autobioluminescent HEK293 cells were performed. H_2_O_2_ levels were measured using the ROS-Glo assay (Promega) according to the manufacturer's instructions. Each plate of the autobioluminescent pCMV_Lux_-expressing or wild type positive control HCT116 and HEK293 cells was incubated at 37°C and 5% CO_2_ for 18 h before the addition of 20 µl 125 µM H_2_O_2_ substrate (provided in the kit), and each well was then incubated for an additional 6 h followed by bioluminescent measurements of H_2_O_2_ levels. Bioluminescent readings were obtained using a SynergyII plate reader (BioTek) with a 1 sec integration time and reported as relative light units. This process was repeated three times to obtain three biological replicates, each consisting of three technical replicates. Relative light units from each plating density were averaged and background values representative of the relative light units detected from the 0 cells/well treatment conditions were subtracted to remove compounding effects from potentially reactive oxygen species in the medium. The average values for each of the triplicate plates were used to determine standard errors of the mean for each assay.

### Cell viability assays

Autobioluminescent HEK293 and HCT116 cells stably transfected with pCMV_Lux_ vectors were seeded at 5000 cell/well in 100 µl medium volumes into six sets of triplicate wells in two separate 96 well plates. Identical platings of untransfected cells were made along with triplicate wells of medium only to serve as positive and negative controls. Each set of triplicate wells for both the pCMV_Lux_-expressing and untransfected control cells were treated with increasing concentrations of the cytotoxic compound Zeocin at 0, 200, 400, 600, 800, and 1000 µg/ml. One plate was then incubated at 37°C and 5% CO_2_ for 24 h before being subjected to viability analysis using the CellTiter 96 Non-Radioactive Cell Proliferation Assay (MTT) (Promega) according to the manufacturer's instructions. Absorbance at 570 nm was determined using a SynergyII plate reader (BioTek) and reported as percentage of absorbance relative to untreated control cells. For all measurements, the background absorbance values derived from the medium only controls were subtracted to provide background corrected averages. The second plate was incubated for 48 h before being processed in an identical fashion. This process was repeated three times to obtain three biological replicates, each consisting of three technical replicates. Absorbance values from each treatment level were averaged and the average values for each of the triplicate plates were used to determine standard errors of the mean for each assay.

### Induction of cytotoxicity by Zeocin treatment

HEK293 and HCT116 cells were seeded at 10000 cell/well in 100 µl medium volumes into four sets of triplicate wells in two separate 96 well plates. On plate was and transfected with pCMV_Lux_, while the second plate was transfected with the pGL4.50/luc2 vector (Promega), which houses a humanized firefly luciferase gene. Identical platings of untransfected cells were included in both plates to serve as negative controls. Twenty hours post transfection, each set of triplicate wells for both the pCMV_Lux_-expressing, pGL4.50/luc2-expressing, and untransfected control cells were treated with increasing concentrations of the cytotoxic compound Zeocin at 0, 200, 400, and 800 µg/ml. Both plates were then incubated at 37°C and 5% CO_2_ for 6 h before being subjected to bioluminescent imaging. Cells transfected with the pCMV_Lux_ vector were imaged in an IVIS Lumina imaging system (PerkinElmer) with a 10 min integration time. Cells transfected with pGL4.50/luc2 vectors were spiked with 300 µg/ml luciferin and immediately imaged using a 1 sec integration. All bioluminescent readings were recorded as percentages of the bioluminescent output of untreated control cells. Additional bioluminescent readings were obtained at 12, 18, and 24 h post Zeocin treatment, with fresh luciferin applied to the pGL4.50/luc2-transfected cells prior to each reading. Following the 24 h bioluminescent reading, all cells were subjected to the CellTiter 96 Non-Radioactive Cell Proliferation Assay (MTT) (Promega) according to the manufacturer's instructions. MTT assay values were obtained as absorbance values at 570 nm using a SynergyII plate reader (BioTek) and reported as percentage of absorbance relative to untreated control cells. For all measurements, the background absorbance values derived from the medium only controls were subtracted to provide background corrected averages. This process was repeated three times to obtain three biological replicates, each consisting of three technical replicates. Absorbance values from each treatment level were averaged and the average values for each of the triplicate plates were used to determine standard errors of the mean for each assay. Pearson correlation coefficients were determined to correlate bioluminescent output levels under each treatment conditions with its paired MTT viability measurements.

### Statistical analysis

Means ±S.E.M. were calculated and significant differences between groups were determined using the Student's *t*-test at *p*≤0.05. All R^2^ values describing the correlations between various treatment effects were determined by calculating Pearson correlation coefficients. All statistical analyses were performed using Microsoft Excel.

## Supporting Information

Figure S1 **Arrangement and function of the bacterial luciferase operon.** The bacterial luciferase operon encodes all required proteins for the establishment of an autobioluminescent phenotype. The *luxA* and *luxB* genes encode for two halves of a heterodimeric luciferase (top, center), the *luxC*, *luxD*, and *luxE* genes encode for a reductase, a synthase, and a transferase, respectively. These gene products form a tetrameric trimer (bottom left) that acts as a cohesive unit to convert and recycle the required aliphatic aldehyde substrate from intracellular components. The *frp* gene, which is not found in all species, encodes a flavin reductase (bottom right) that shifts the intracellular FMN:FMNH_2_ balance to a more reduced state in order to supply the remaining FMNH_2_ co-substrate. When expressed concurrently, this allows for the continuous generation of bioluminescence without the need for any external input.
(TIF)

Figure S2 **Transcript levels for each of the 2A-linked** ***lux*** **genes were greater when placed under control of the CMV promoter relative to EF1α-mediated expression in HEK293 cells.** When normalized to *β*-actin, CMV-driven expression lead to 5- to 10-fold increases in transcript abundance compared to an otherwise identical expression system driven by EF1α.
(TIF)

Figure S3 **Detection of autobioluminescence from HEK293 cells stably expressing the pCMV_Lux_ plasmid.** (a) Representation of autobioluminescent detection at a 1 sec acquisition time using either a PMT-based SynergyII plate reader (graph) or an IVIS Lumina CCD camera (inset image). CCD camera acquired images were similar across all assayed time points. (b) Using the more sensitive CCD camera-based imaging approach, the minimum population size required to produce a signal significantly above background noise detection (*p*≤0.05) was determined to be 1,000 cells.
(TIF)

Figure S4 **Autobioluminescent output correlates strongly with cellular population size across all cell types surveyed.**
(TIF)

Figure S5 **Correlation of metabolic activity levels in wild type and autobioluminescent cells.** Correlation of measured relative light units from the CellTiter-Glo assay comparing ATP levels between identical numbers of plated HEK293 (a) and HCT116 (b) cells. Increases in ATP levels in the 24 h samples relative to 0 h samples are due to increases in total cell numbers resulting from cellular growth.
(TIFF)

Figure S6 **Correlation of reactive oxygen species levels in wild type and autobioluminescent cells.** Correlation of measured relative light units from the ROS-Glo assay comparing H_2_O_2_ levels between identical numbers of plated wild type (X – axis) and autobioluminescent (Y – axis) HEK293 (a) and HCT116 (b) cells. Increasingly negative values for the HEK293 cells indicate an increasing removal of reactive oxygen species obtained from the culture medium as larger cell numbers are plated. Vertical error bars represent S.E.M. of triplicate assays conducted on autobioluminescent cell lines. Horizontal error bars represent S.E.M. of triplicate assays conducted on wild type cell lines. Negative values denote a reduction in ROS availability relative to background ROS availability stemming from components in the cell culture medium.
(TIF)

Figure S7 **Comparison of treatment-induced cytotoxic effects between wild type and autobioluminescent cell lines.** Wild type and autobioluminescent HEK293 were screened for total cellular viability using a standard MTT assay at 24 (a) and 48 h (b) under increasing levels of cytotoxic Zeocin treatment. Wild type and autobioluminescent HCT116 were similarly tested at 24 (c) and 48 h (d). Values are reported as percent absorbance of MTT assay results as compared to untreated control cells, with higher percentages representing increasing numbers of viable cells.
(TIF)

Figure S8 **Comparison of treatment-induced cytotoxic effects between autobioluminescent and firefly luciferase-expressing cell lines.** Bioluminescent signals from autobioluminescent and *luc*-expressing HCT116 (a) and HEK293 (b) cells exposed to increasing levels of cytotoxic Zeocin treatment were correlated with viability readings from standard MTT assays.
(TIF)

Table S1 **Viral 2A elements used in this study.** The viral 2A elements used to create a functional *lux* operon for expression in human cells. Glycine/serine flexible linkers are in lowercase, conserved proline/glycine/proline motifs are highlighted, with autocleavage occurring between the glycine and final proline.
(PDF)

Table S2 **Transcript levels of 2A-linked** ***lux*** **genes under control of the CMV and EF1α promoters.**
(PDF)

Table S3 **Comparison of the metabolic activity levels of autobioluminescent cells relative to similarly treated wild type controls.** *p* values of Students T-Tests between measured ATP concentrations of wild type and autobioluminescent HEK293 and HCT116 cells at 0 and 24 h post plating. Significant differences in ATP levels (*p*≤0.05) are highlighted in green, while statistically similar ATP levels (*p*>0.05) are highlighted in red.
(PDF)

Table S4 D**etection of cytotoxicity via reductions in bioluminescent output.** *p* values of Student's *t*-tests between Zeocin-treated *lux* and *luc* expressing HEK293 and HCT116 cells and untreated control cells. Statistically significant decreases in bioluminescent output (*p*≤0.05) are highlighted in green, while statistically similar bioluminescent output levels (*p*>0.05) are highlighted in red.
(PDF)

Table S5 **Transcript levels of 2A-linked** ***lux*** **genes following exposure to 100 ng doxycycline/ml.**
(PDF)
