# Supplementary figures and images for: Expression of a Humanized Viral 2A-Mediated lux Operon Efficiently Generates Autonomous Bioluminescence in Human Cells
Source: PLoS One. 2014 May 2;9(5):e96347. doi: 10.1371/journal.pone.0096347 (PMC4008522; doi:10.1371/journal.pone.0096347)

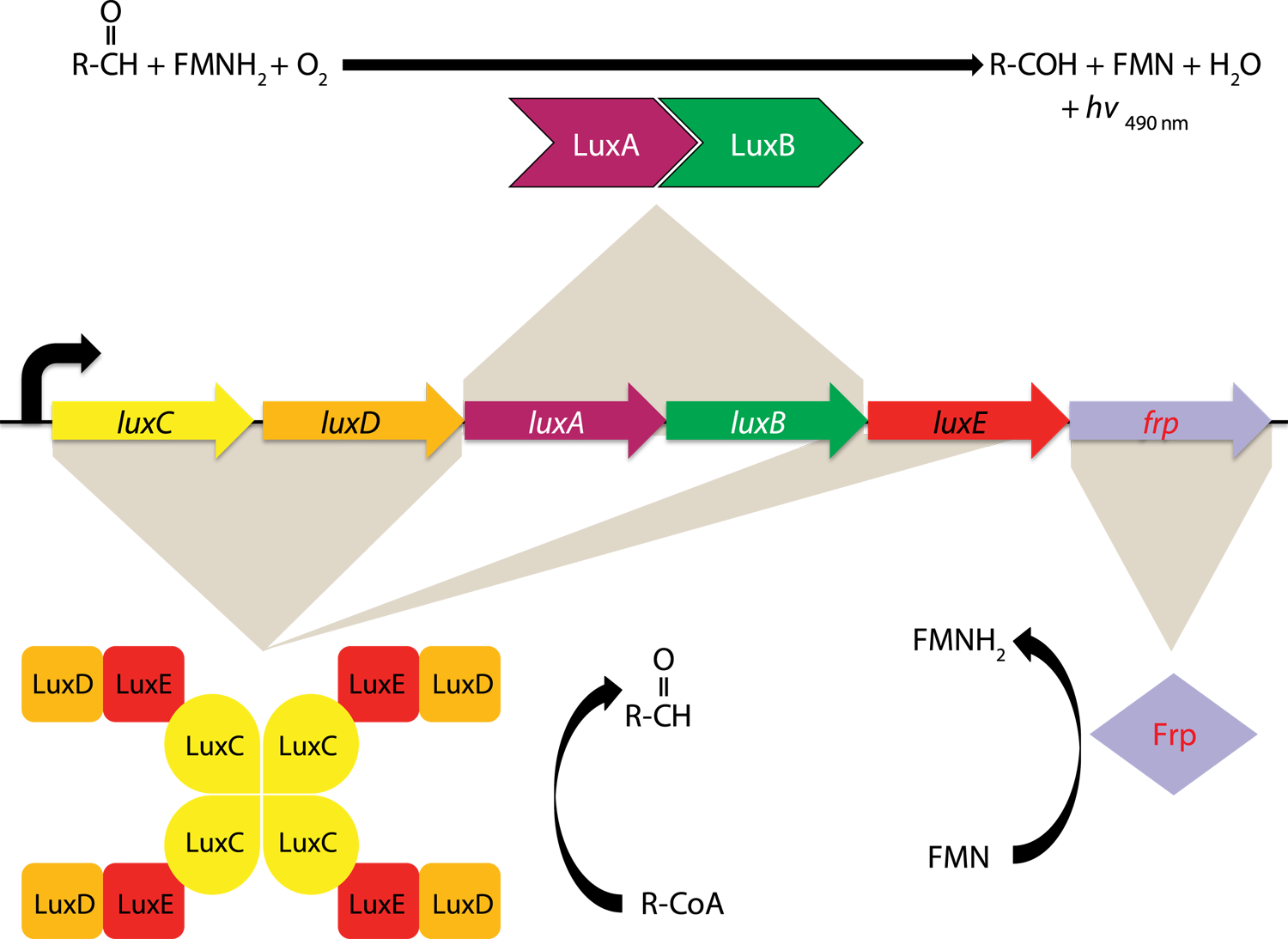

Supplement: Figure S1 — Arrangement and function of the bacterial luciferase operon. The bacterial luciferase operon encodes all required proteins for the establishment of an autobioluminescent phenotype. The luxA and luxB genes encode for two halves of a heterodimeric luciferase (top, center), the luxC, luxD, and luxE genes encode for a reductase, a synthase, and a transferase, respectively. These gene products form a tetrameric trimer (bottom left) that acts as a cohesive unit to convert and recycle the required aliphatic aldehyde substrate from intracellular components. The frp gene, which is not found in all species, encodes a flavin reductase (bottom right) that shifts the intracellular FMN:FMNH2 balance to a more reduced state in order to supply the remaining FMNH2 co-substrate. When expressed concurrently, this allows for the continuous generation of bioluminescence without the need for any external input. (TIF) [file pone.0096347.s001.tif]

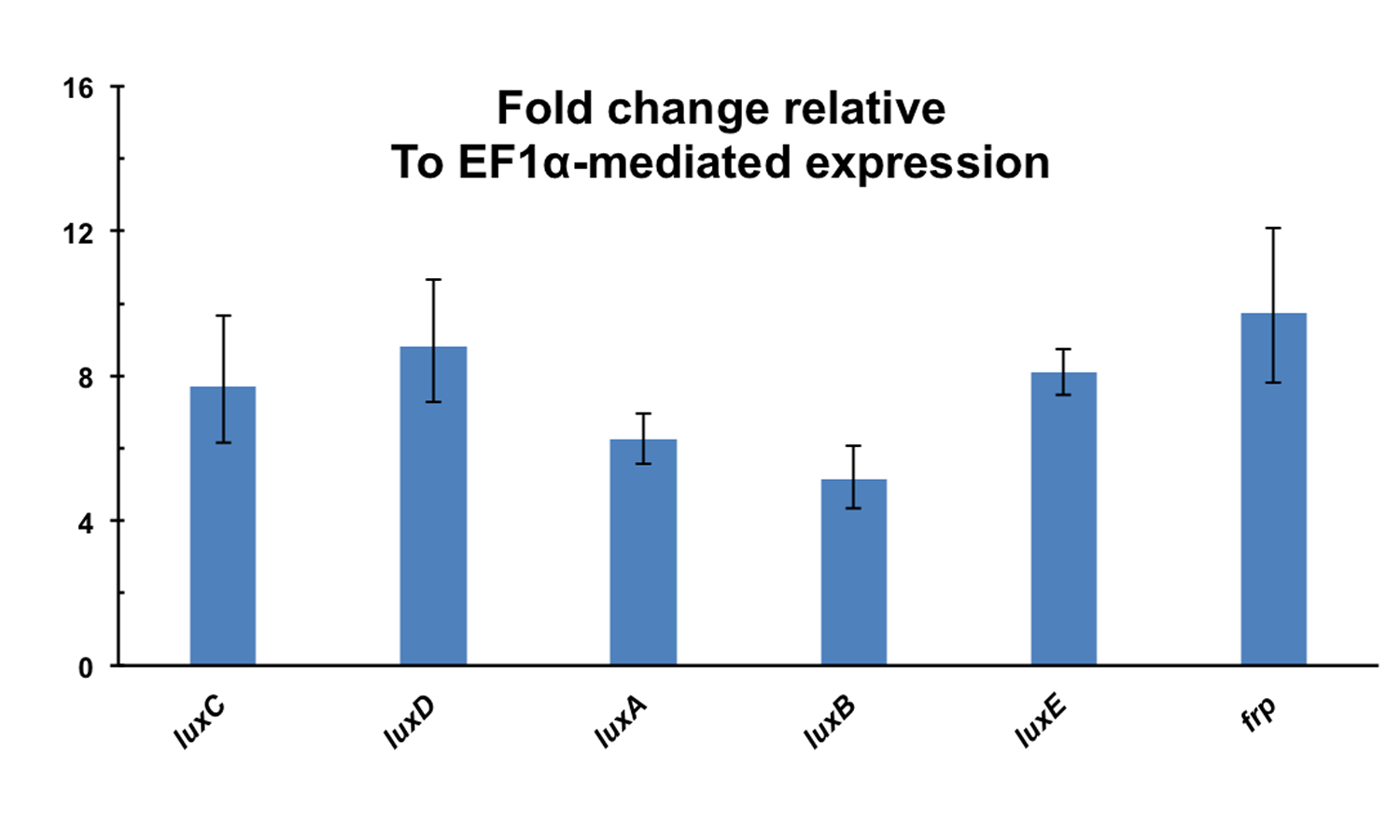

Supplement: Figure S2 — Transcript levels for each of the 2A-linked lux genes were greater when placed under control of the CMV promoter relative to EF1α-mediated expression in HEK293 cells. When normalized to β-actin, CMV-driven expression lead to 5- to 10-fold increases in transcript abundance compared to an otherwise identical expression system driven by EF1α. (TIF) [file pone.0096347.s002.tif]

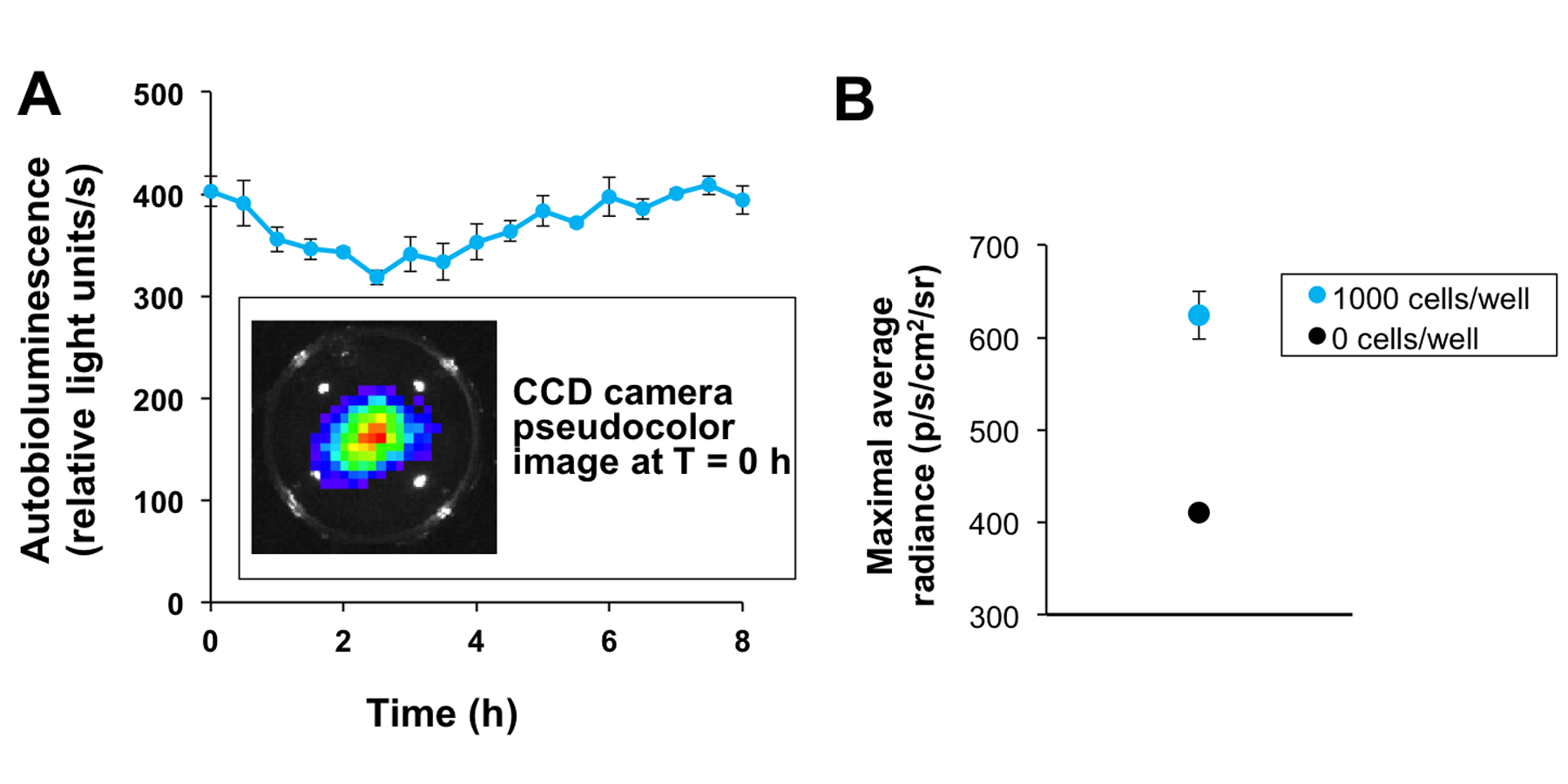

Supplement: Figure S3 — Detection of autobioluminescence from HEK293 cells stably expressing the pCMVLux plasmid. (a) Representation of autobioluminescent detection at a 1 sec acquisition time using either a PMT-based SynergyII plate reader (graph) or an IVIS Lumina CCD camera (inset image). CCD camera acquired images were similar across all assayed time points. (b) Using the more sensitive CCD camera-based imaging approach, the minimum population size required to produce a signal significantly above background noise detection (p≤0.05) was determined to be 1,000 cells. (TIF) [file pone.0096347.s003.tif]

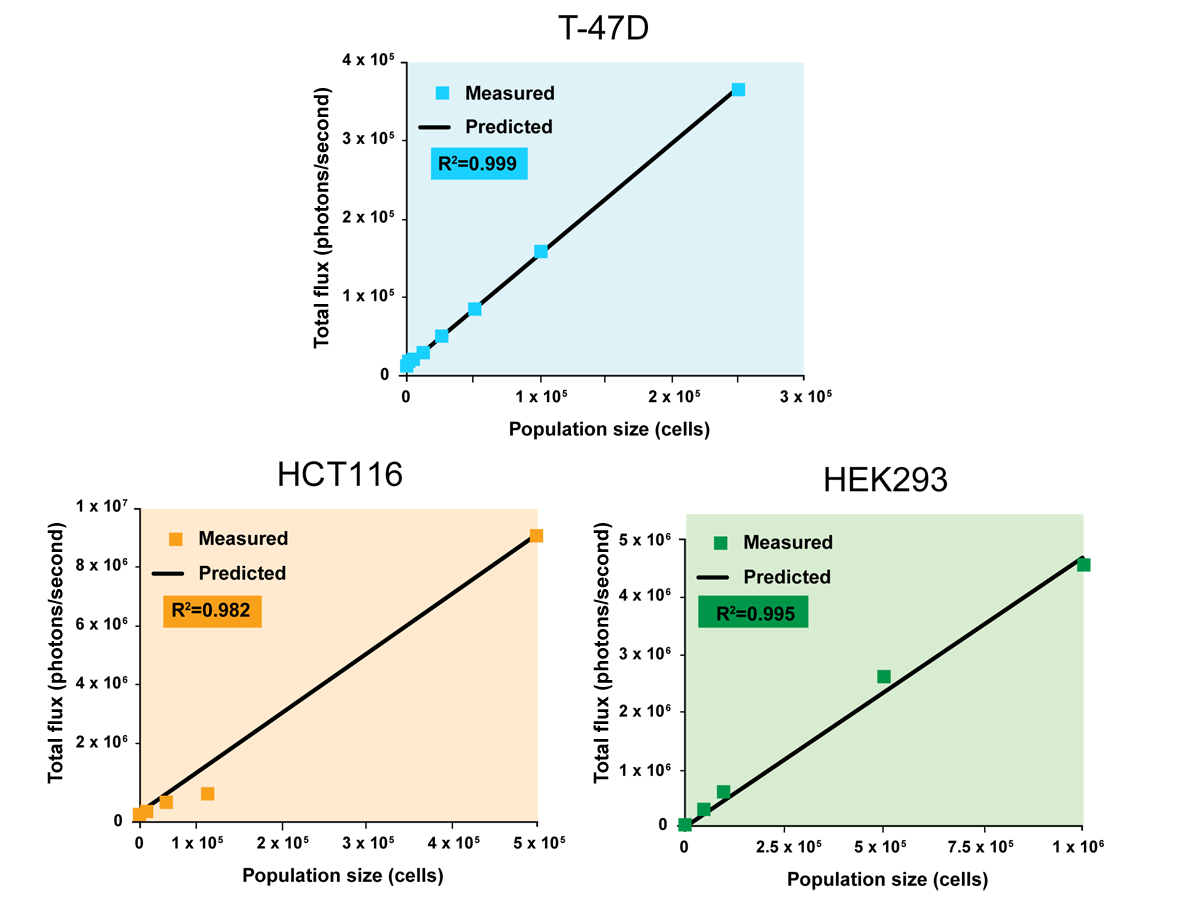

Supplement: Figure S4 — Autobioluminescent output correlates strongly with cellular population size across all cell types surveyed. (TIF) [file pone.0096347.s004.tif]

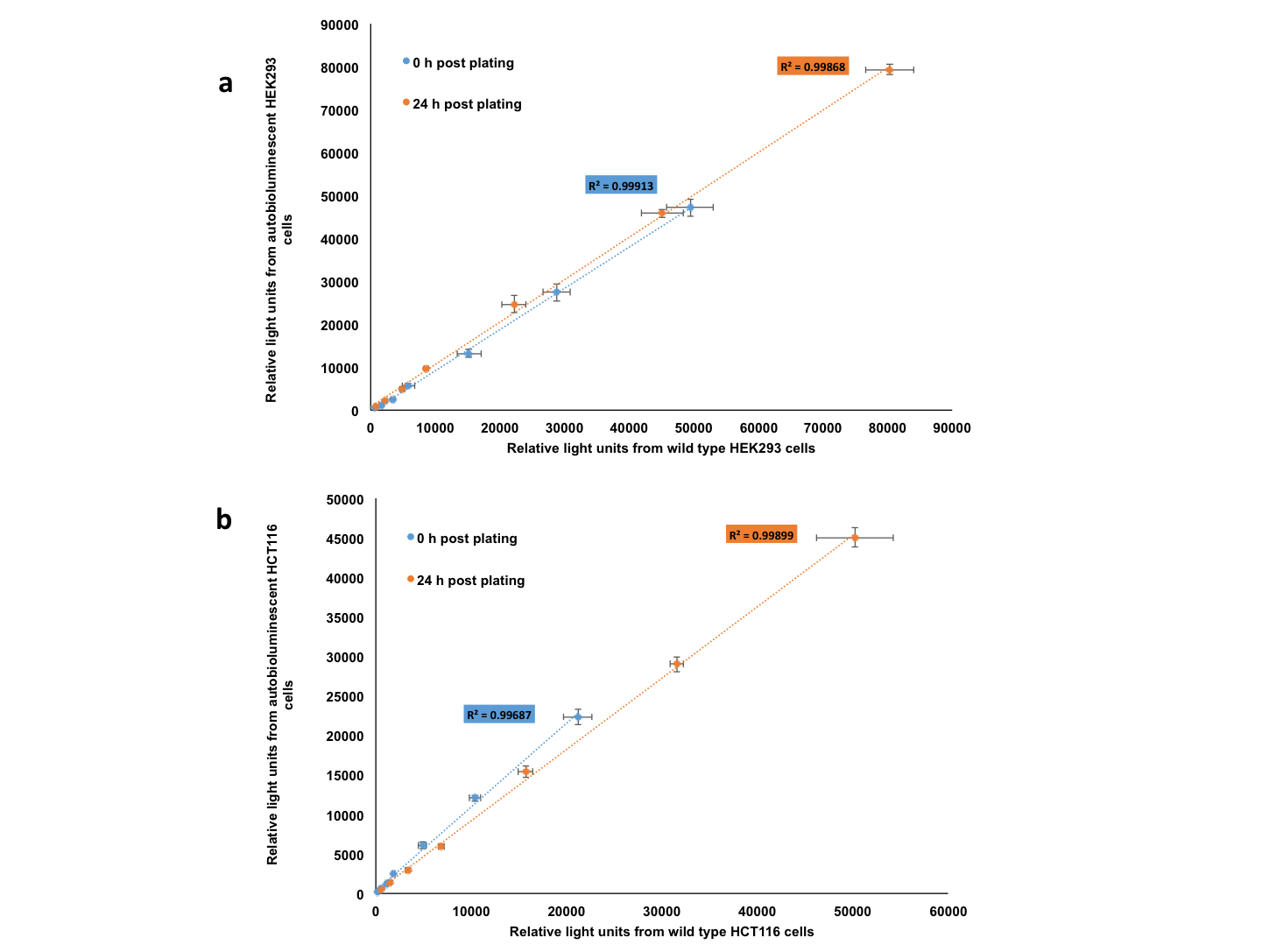

Supplement: Figure S5 — Correlation of metabolic activity levels in wild type and autobioluminescent cells. Correlation of measured relative light units from the CellTiter-Glo assay comparing ATP levels between identical numbers of plated HEK293 (a) and HCT116 (b) cells. Increases in ATP levels in the 24 h samples relative to 0 h samples are due to increases in total cell numbers resulting from cellular growth. (TIFF) [file pone.0096347.s005.tiff]

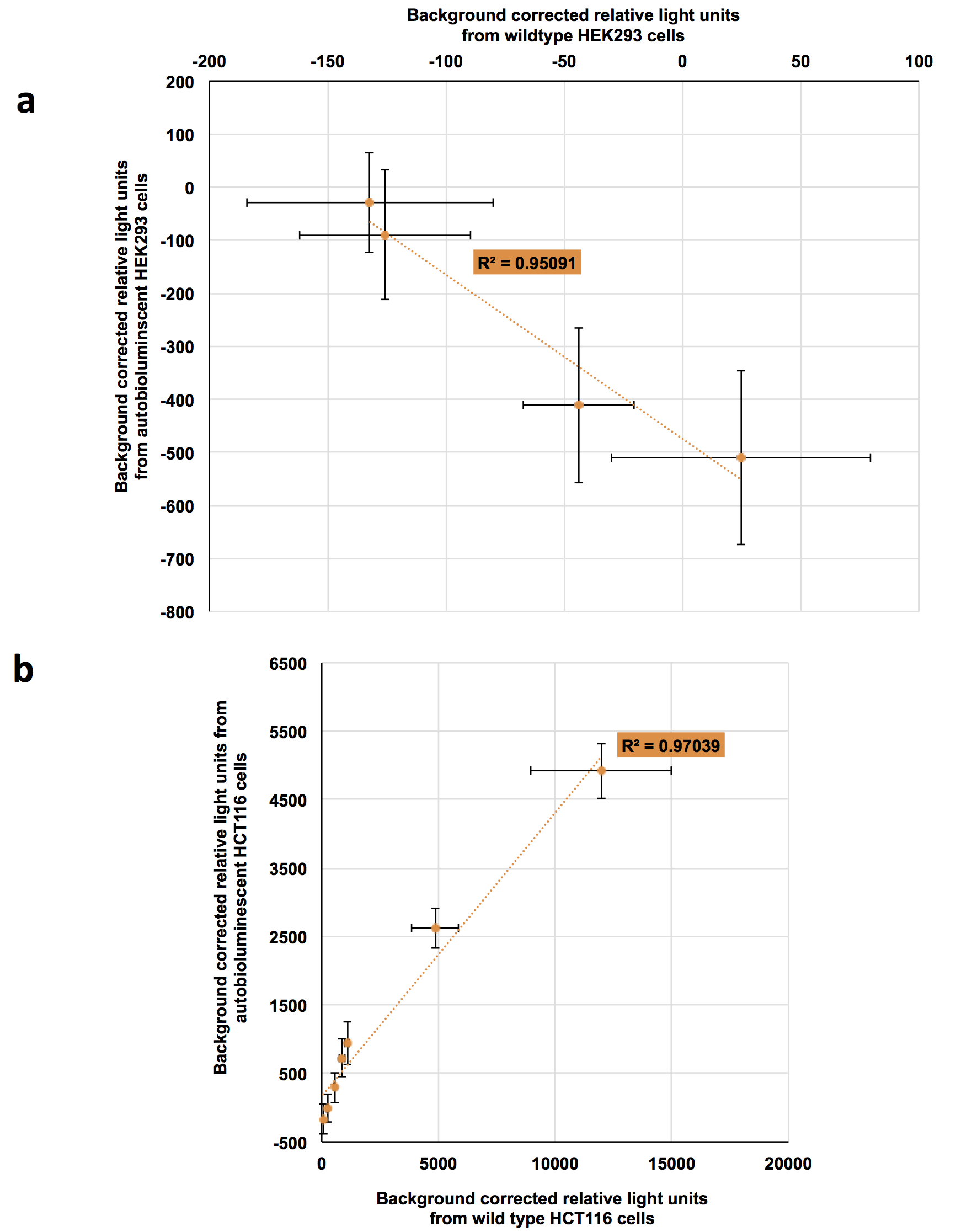

Supplement: Figure S6 — Correlation of reactive oxygen species levels in wild type and autobioluminescent cells. Correlation of measured relative light units from the ROS-Glo assay comparing H2O2 levels between identical numbers of plated wild type (X – axis) and autobioluminescent (Y – axis) HEK293 (a) and HCT116 (b) cells. Increasingly negative values for the HEK293 cells indicate an increasing removal of reactive oxygen species obtained from the culture medium as larger cell numbers are plated. Vertical error bars represent S.E.M. of triplicate assays conducted on autobioluminescent cell lines. Horizontal error bars represent S.E.M. of triplicate assays conducted on wild type cell lines. Negative values denote a reduction in ROS availability relative to background ROS availability stemming from components in the cell culture medium. (TIF) [file pone.0096347.s006.tif]

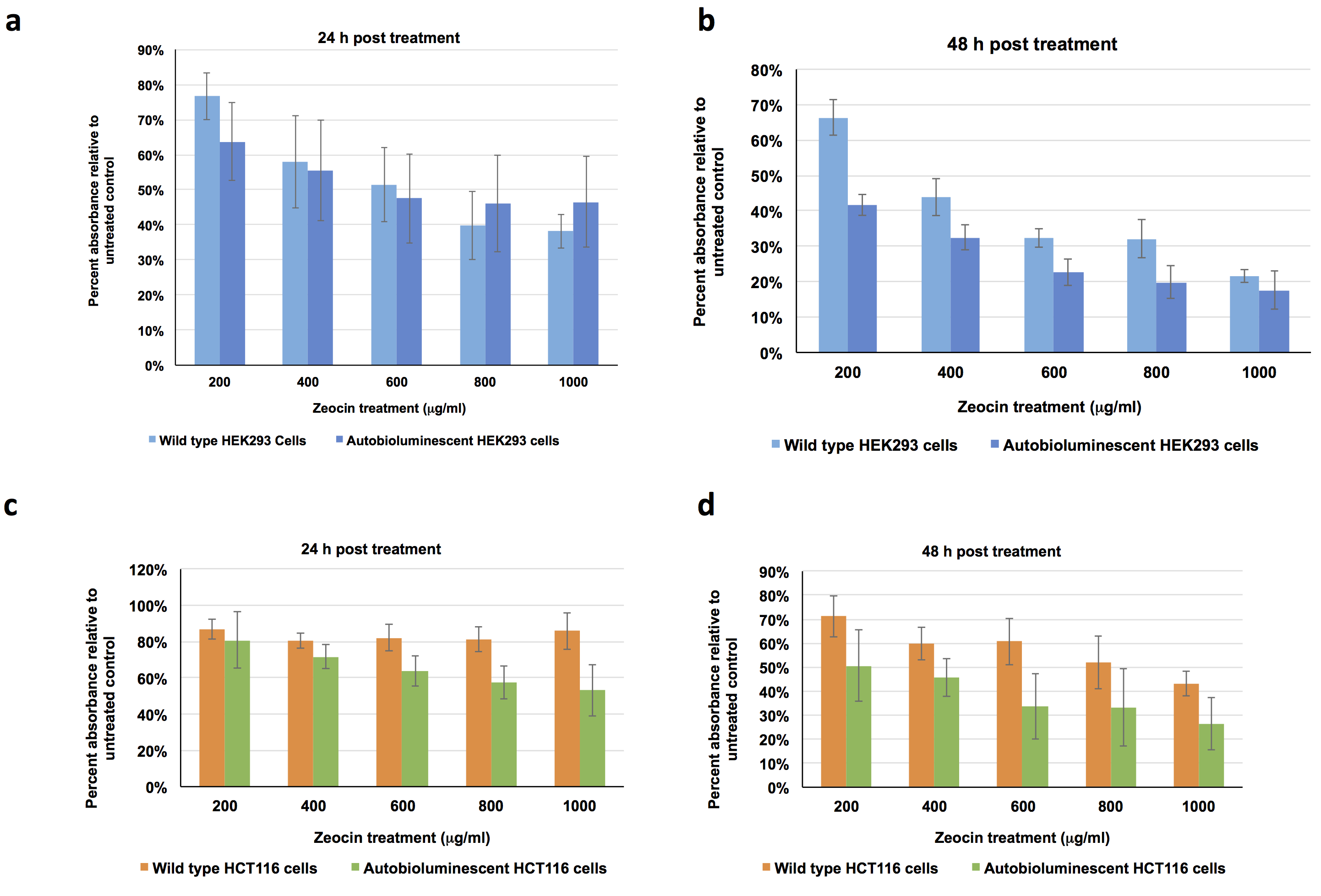

Supplement: Figure S7 — Comparison of treatment-induced cytotoxic effects between wild type and autobioluminescent cell lines. Wild type and autobioluminescent HEK293 were screened for total cellular viability using a standard MTT assay at 24 (a) and 48 h (b) under increasing levels of cytotoxic Zeocin treatment. Wild type and autobioluminescent HCT116 were similarly tested at 24 (c) and 48 h (d). Values are reported as percent absorbance of MTT assay results as compared to untreated control cells, with higher percentages representing increasing numbers of viable cells. (TIF) [file pone.0096347.s007.tif]

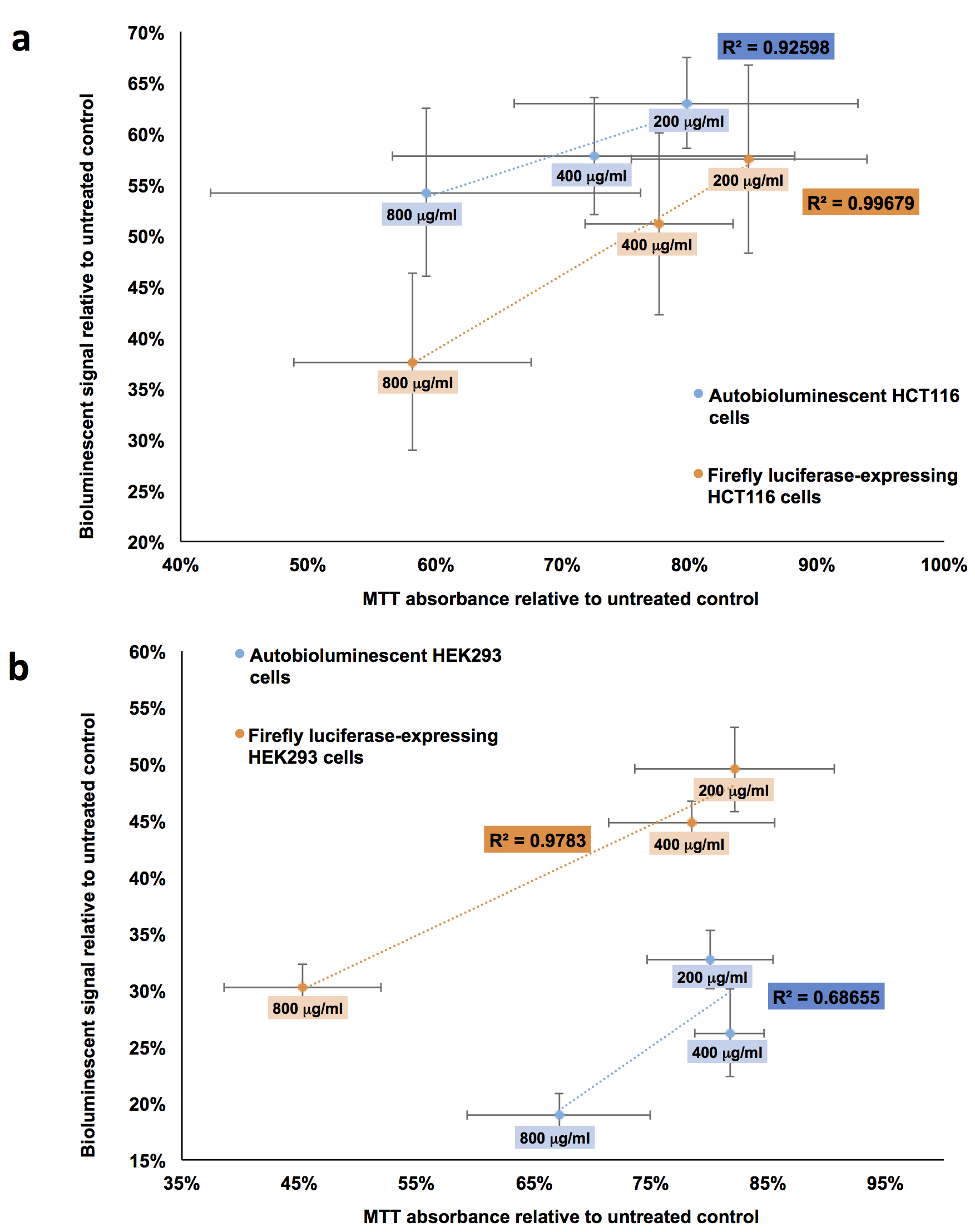

Supplement: Figure S8 — Comparison of treatment-induced cytotoxic effects between autobioluminescent and firefly luciferase-expressing cell lines. Bioluminescent signals from autobioluminescent and luc-expressing HCT116 (a) and HEK293 (b) cells exposed to increasing levels of cytotoxic Zeocin treatment were correlated with viability readings from standard MTT assays. (TIF) [file pone.0096347.s008.tif]
