# Supplementary material for: Expression of a Humanized Viral 2A-Mediated lux Operon Efficiently Generates Autonomous Bioluminescence in Human Cells
Source: PLoS One. 2014 May 2;9(5):e96347. doi: 10.1371/journal.pone.0096347 (PMC4008522; doi:10.1371/journal.pone.0096347)
Supplement: Table S1 — Viral 2A elements used in this study. The viral 2A elements used to create a functional lux operon for expression in human cells. Glycine/serine flexible linkers are in lowercase, conserved proline/glycine/proline motifs are highlighted, with autocleavage occurring between the glycine and final proline. (PDF) [file pone.0096347.s009.pdf]

Table S1

Viral 2A elements used in this study

Name	Source	Sequence	Translation
F-2A	Foot and mouth disease virus	ggtggcggatcaggcggtgggCAGCTGTTGAA<br>TTTTGACCTTCTCAAGTTGGCGGGA<br>GACGTGGAGTCCAACCCAGGGCCC	g g g s g g g Q L L N F D L L K L A G D V E S N P G P
E-2A	Equine rhinitis A virus	gggggaggttcaggagggggcCAGTGTACTA<br>ACTACGCTTTGTTGAAACTCGCTGG<br>CGATGTTGAAAGTAACCCCGGTCCT	g g g s g g g Q C T N Y A L L K L A G D V E S N P G P
Ta-2A	*Thosea asigna* virus (synthetic sequence)	ggtggcggttcaggcggaggaGAAGGTAGGG<br>GTTCATTATTGACCTGTGGAGATGT<br>CGAAGAAAACCCAGGACCC	g g g s g g g E G R G S L L T C G D V E E N P G P
P-2A	*Porcine teschovirus* 1	ggtgggggatcagggggtggcGCGACAAACT<br>TTAGCTTGCTGAAGCAAGCTGGTGA<br>CGTTGAGGAGAATCCCGGACCA	g g g s g g g A T N F S L L K Q A G D V E E N P G P
T-2A	*Thosea asigna* virus	ggtggaggcagcgggggcggtGAGGGCAGAG<br>GAAGTCTTCTAACATGCGGTGACGT<br>GGAGGAGAATCCCGGCCCT	g g g s g g g E G R G S L L T C G D V E E N P G P

The viral 2A elements used to create a functional *lux* operon for expression in human cells. Glycine/serine flexible linkers are in lowercase, conserved proline/glycine/proline motifs are highlighted, with autocleavage occurring between the glycine and final proline.
