# Supplementary material for: Expression of a Humanized Viral 2A-Mediated lux Operon Efficiently Generates Autonomous Bioluminescence in Human Cells
Source: PLoS One. 2014 May 2;9(5):e96347. doi: 10.1371/journal.pone.0096347 (PMC4008522; doi:10.1371/journal.pone.0096347)
Supplement: Table S2 — Transcript levels of 2A-linked lux genes under control of the CMV and EF1α promoters. (PDF) [file pone.0096347.s010.pdf]

Table S2

Transcript levels of 2A-linked *lux* genes under control of the CMV and EF1α promoters.

**Gene**	**CMV-directed expression relative to EF1α-directed expression ($2^{-\Delta\Delta Cq}$)**	**Range (calculated based on standard error of ΔΔCq)**
luxC	7.71	6.16 - 9.65
luxD	8.80	7.27 - 10.66
luxA	6.24	5.57 - 10.66
luxB	5.14	4.35 - 6.07
luxE	8.09	7.48 - 8.75
frp	9.72	7.82 - 12.10
