# Supplementary material for: Expression of a Humanized Viral 2A-Mediated lux Operon Efficiently Generates Autonomous Bioluminescence in Human Cells
Source: PLoS One. 2014 May 2;9(5):e96347. doi: 10.1371/journal.pone.0096347 (PMC4008522; doi:10.1371/journal.pone.0096347)
Supplement: Table S3 — Comparison of the metabolic activity levels of autobioluminescent cells relative to similarly treated wild type controls. p values of Students T-Tests between measured ATP concentrations of wild type and autobioluminescent HEK293 and HCT116 cells at 0 and 24 h post plating. Significant differences in ATP levels (p≤0.05) are highlighted in green, while statistically similar ATP levels (p>0.05) are highlighted in red. (PDF) [file pone.0096347.s011.pdf]

Table S3

Comparison of the metabolic activity levels of autobioluminescent cells relative to similarly treated wild type controls.

Number of Cells Plated	Autobioluminescent HEK293 0 h Post Plating	Autobioluminescent HEK293 24 h Post Plating	Autobioluminescent HCT116 0 h Post Plating	Autobioluminescent HCT116 24 h Post Plating
**100**	0.21	0.41	0.37	0.14
**250**	0.10	0.77	0.32	0.71
**500**	0.07	0.90	0.72	0.25
**1000**	0.95	0.04	0.02	0.07
**2500**	0.39	0.41	0.14	0.78
**5000**	0.66	0.86	0.08	0.10
**10000**	0.60	0.80	0.55	0.29

p values of Student's t-tests between measured ATP concentrations in autobioluminescent HEK293 and HCT116 cells at 0 and 24 h post plating relative to similarly treated wild type control cells. Significant differences in ATP levels ($p \leq 0.05$) are highlighted in green, while statistically similar ATP levels ($p > 0.05$) are highlighted in red.
