# Supplementary material for: Expression of a Humanized Viral 2A-Mediated lux Operon Efficiently Generates Autonomous Bioluminescence in Human Cells
Source: PLoS One. 2014 May 2;9(5):e96347. doi: 10.1371/journal.pone.0096347 (PMC4008522; doi:10.1371/journal.pone.0096347)
Supplement: Table S4 — Detection of cytotoxicity via reductions in bioluminescent output. p values of Student's t-tests between Zeocin-treated lux and luc expressing HEK293 and HCT116 cells and untreated control cells. Statistically significant decreases in bioluminescent output (p≤0.05) are highlighted in green, while statistically similar bioluminescent output levels (p>0.05) are highlighted in red. (PDF) [file pone.0096347.s012.pdf]

**Table S4**

Detection of cytotoxicity via reductions in bioluminescent output.

| | Hours Post Treatment | Treatment Level: 200 µg/ml | 400 µg/ml | 800 µg/ml |
|---|---|---|---|---|
| **Autobioluminescent HEK293 Cells** | **0** | 0.31 | 0.15 | 0.31 |
| | **6** | 0.13 | 0.21 | 0.14 |
| | **12** | 0.05 | 0.07 | 0.05 |
| | **18** | 0.02 | 0.02 | 0.02 |
| | **24** | 0.05 | 0.05 | 0.04 |
| ***luc*-Expressing HEK293 Cells** | **0** | 0.46 | 0.40 | 0.37 |
| | **6** | 0.04 | 0.35 | 0.17 |
| | **12** | 0.14 | 0.17 | 0.13 |
| | **18** | 0.01 | 0.04 | 0.02 |
| | **24** | 0.02 | 0.02 | 0.01 |
| **Autobioluminescent HCT116 Cells** | **0** | 0.77 | 0.81 | 0.53 |
| | **6** | 0.28 | 0.24 | 0.24 |
| | **12** | 0.16 | 0.12 | 0.12 |
| | **18** | 0.10 | 0.10 | 0.11 |
| | **24** | 0.12 | 0.12 | 0.14 |
| ***luc*-Expressing HCT116 Cells** | **0** | 0.94 | 0.31 | 0.77 |
| | **6** | 0.46 | 0.30 | 0.22 |
| | **12** | 0.08 | 0.08 | 0.07 |
| | **18** | 0.02 | 0.01 | 0.02 |
| | **24** | 0.02 | 0.01 | 0.00 |

*p* values of Student's *t*-tests between Zeocin-treated *lux* and *luc* expressing HEK293 and HCT116 cells and untreated control cells. Statistically significant decreases in bioluminescent output ($p \leq 0.05$) are highlighted in green, while statistically similar bioluminescent output levels ($p > 0.05$) are highlighted in red.
