# Supplementary material for: Expression of a Humanized Viral 2A-Mediated lux Operon Efficiently Generates Autonomous Bioluminescence in Human Cells
Source: PLoS One. 2014 May 2;9(5):e96347. doi: 10.1371/journal.pone.0096347 (PMC4008522; doi:10.1371/journal.pone.0096347)
Supplement: Table S5 — Transcript levels of 2A-linked lux genes following exposure to 100 ng doxycycline/ml. (PDF) [file pone.0096347.s013.pdf]

Table S5

Transcript levels of 2A-linked *lux* genes following exposure to 100 ng doxycycline/ml.

**Gene**	**Fold change ($2^{-\Delta\Delta Cq}$)**	**Range (calculated based on standard error of ΔΔCq)**
luxC	9.26	7.63 - 11.22
luxD	8.20	6.86 - 9.80
luxA	7.77	6.29 - 9.59
luxB	7.04	5.88 - 8.44
luxE	7.26	5.48 - 9.62
frp	5.57	4.36 - 7.12
